# Change and persistence in healthcare inequities: Access to elective surgery in Finland in 1992–2003

**DOI:** 10.1177/1403494808098505

**Published:** 2010-03

**Authors:** Kristiina Manderbacka, Martti Arffman, Alastair Leyland, Alison McCallum, Ilmo Keskimäki

**Affiliations:** 1 Health Services Research, STAKES (National Research and Development Centre for Welfare and Health), Helsinki, Finland; 2 MRC Social and Public Health Science Unit, Glasgow, UK; 3 NHS Lothian Health Board, Edinburgh, UK

**Keywords:** Elective surgery, health services research, hospital use, socioeconomic equity

## Abstract

*Aims:* Many countries experience persistent or increasing socioeconomic disparities in specialist care. This study examines the socioeconomic distribution of elective surgery from 1992 to 2003 in Finland. *Methods:* Administrative registers were used to identify common elective procedures performed in all public and private hospitals in Finland in 1992–2003. Patients’ individual sociodemographic data came from 1990–2003 census and employment statistics databases. First coronary revascularisation, hip and knee replacement, lumbar disc operation, cataract extraction, hysterectomy and prostatectomy on residents aged 25–84 years were analysed. Age-standardized procedure rates by income quintile were calculated for both genders, and concentration indices were developed and applied to age-standardized procedure rates in 5% income groups for each study year. *Results:* Most procedure rates increased during the study period. Three trends emerged: declining inequality for coronary revascularisations, an increase and then a decline in cataract extractions and primary knee replacements among men, and positive relationships between income and treatment for hysterectomy and lumbar disc operations. ***Conclusions:* Our results suggest that structural features – uneven availability, co-payments and plurality of provision – sustain inequity in access; decreasing inequities reflect directed service expansion. Increased attention to collective, prospective funding of primary and specialist ambulatory care is required to increase equity of access to elective surgery.**

## Background

Equity in health and healthcare are considered to be important goals for health policy in Finland, as in other industrialized countries. Despite universal access to healthcare having been enshrined in law for decades, many countries, including Finland, continue to report socioeconomic inequalities in treatment rates in ambulatory care [1]. In specialist care, similar results have been reported in Finland. In an earlier study [2], covering 1988 and 1996, overall hospital use was found to be greater in lower socioeconomic groups than among better-off groups. However, affluent patients underwent common, planned surgical procedures more frequently. A general trend of increasing disparities was found in the content of care both overall and in individual procedures and surgical diagnostic categories from 1988 to 1996. Similar but narrowing disparities have been reported in coronary revascularisations; in revascularisations, a large increase in operation rates took place in this time period [3]. Similar results on disparities in elective operations have also been reported from other countries with similar healthcare systems, e.g. in terms of hip [4] and knee replacements [5] and coronary revascularisations [6,7].

This article examines elective surgery as a case study for access to specialized hospital treatment from 1992 to 2003. Elective surgery is a useful exemplar for studying access to specialist care, since it exhibits a strong element of discretion on the part of health service providers as to how and when treatment is offered.

## Materials and method

### Data

Hospital data from 1992 to 2003 were used to assess changes in the socioeconomic distribution in the use of common surgical procedures. Data on selected elective procedures were obtained from the Finnish Care Register, which covers all hospital discharges in all public and private hospitals in Finland. The study population consisted of all people resident in Finland between 1992 and 2003 aged 25–84 years at the beginning of each year.

Seven procedures were selected to study socioeconomic differences in rates of common and usually elective surgical procedures: coronary revascularisation, primary hip replacement operation, primary knee replacement operation, lumbar disc operation, hysterectomy, prostatectomy, and cataract operation. Coronary revascularisations included coronary artery bypass grafting for the whole study period, as well as percutaneous transluminal coronary angioplasties (PTCAs) from 1994 onwards. PTCAs performed in 1992–93 were missing, since they were not recorded in the Finnish Care Register before 1994. According to statistics from the Finnish Heart Association, PTCAs covered approximately one-quarter of revascularisations during those 2 years [8]. For hip replacements, those performed in the context of a fracture of the femur (ICD 10 code S72, ICD 9 codes 820–821) were excluded. Surgical operations were coded according to the classification of procedures of the Finnish Hospital League [9] until 1996, and thereafter according to the NOMESCO classification [10]. Since cataract operations can be performed in private outpatient clinics, and therefore not be included in the discharge records of the Finnish Care register, the data were complemented with information on operations from the Social Insurance Institution register for reimbursed healthcare use. Additionally, since subsequent operations are likely to be related to preceding ones and thus cannot be considered as independent observations, we focused on first operations. We considered that first operations best illustrate access to, and selection for, hospital care. Access to subsequent operations is more likely to reflect assessed clinical need and willingness to remain engaged with services, factors that require separate analyses.

The hospital data were individually linked to sociodemographic data from Statistics Finland using the personal identification code unique to each resident in Finland. Each record was linked with information referring to 31 December of the preceding year. Persons who were not permanent residents of Finland and those under 25 or over 84 years of age at the beginning of the entry year were excluded from the data. Year of procedure was approximated by the date of hospital admission, since the exact date of procedure event could not be specified. Age was defined as on 31 December of the year before the data entry and classified into 5-year age bands in order to match the age groups in population at risk tables. Family disposable income from the year preceding data entry was derived from the employment statistics, and adjusted for family size using the Organisation for Economic Co-operation and Development (OECD) equivalence scale. The study population was classified into income groups according to family disposable income, based on limits derived from the population at risk tables. Patients who were in long-term institutions were excluded from the data, since family income cannot be determined for this group reliably from the registers used.

For statistical analyses, the population at risk was defined as the resident Finnish population aged 25–84 years. Tabulated data on the population at risk was derived from 1995 census data and the employment statistics for the study years by each of the sociodemographic variables used in the study. This study was approved by the STAKES research ethics committee, the data protection measures were agreed with Statistics Finland, as the proper statistical authority, and the data linkages were considered to be appropriate by the office of the Finnish data protection ombudsman.

### Statistical methods

Annual age-standardized rates for first elective surgical procedures were calculated for men and women separately, using the direct method of standardisation. The resident Finnish population from 2003 was used as the standard population.

In order to explore the socioeconomic distribution of different aspects of hospital care, the distributions of elective operations by income-20ths were analysed using the concentration curves of hospital utilisation. Concentration indices (CIs) were determined to quantify the degree of income-related inequality in these operations. Annual age-standardized CIs were calculated for each elective procedure, separately for men and women. We used the approach of calculating CIs for grouped data presented by Kakwani et al. [11] and van Doorslaer et al. [12]. CIs were calculated as ${\overset{\wedge }{\beta }}_{1}$, the OLS estimator of β_1_, from the regression equation 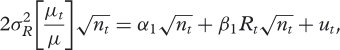 where the subscript *t* = 1, 2, … , *T* is net income-20th (*T* = 20), *R*^t^ is the relative rank of income group *t*, $\sigma_{R}^{2}$ is the variance of the ranks, and μ *_t_* and μ represent age-standardized procedure rates for income group *t* and the mean standardized rate, respectively, with *n_t_* – the person years in income groups – used as weights. As the direct method of standardisation was used, the standard error for the CI was calculated as presented by Kakwani et al. [11]. Average annual percentage changes were calculated for age-standardized operation rates. Changes in the socioeconomic distribution of elective surgical procedures were analysed by estimating linear trends from linear regression models for annual CIs, taking into account the uncertainty by using the inverse of the standard errors as weights. Statistical analyses were performed using the SAS system for Windows, release 9.1.3.

## Results

Procedure rates tended to increase during the study period, especially for coronary revascularisations, primary knee replacement operations and cataract operations, both among men and among women (Table I), with rates for these operations doubling during the study period. The rates for primary hip replacement operations increased more modestly. Lumbar disc operation, hysterectomy and prostatectomy rates increased in the beginning of the study period, but decreased towards the late 1990s and early 2000s. Revascularisation rates were much higher among men than among women, hip replacement operations were relatively evenly distributed, and primary knee operations were almost twice as frequent among women during most of the study years.

**Table I:** Age-standardized procedure rates for men and women in 1992–2003 per 100,000 population.

	1992	1993	1994	1995	1996	1997	1998	1999	2000	2001	2002	2003	Average annual change (%)
Men													
Cataract operation	347	359	402	408	471	494	505	523	508	510	554	584	4.1
Revascularisation	178	208	305	294	320	316	311	311	329	329	347	390	4.0
Lumbar disc operation	102	111	119	111	116	112	109	106	104	91	92	86	– 2.0
Primary hip replacement	96	107	111	102	110	107	103	102	108	113	122	133	2.0
Primary knee replacement	36	40	47	45	54	63	60	64	74	82	89	106	9.3
Prostatectomy	348	372	361	335	336	313	289	280	267	246	252	255	– 3.6
Women													
Cataract operation	451	472	526	517	627	678	681	675	667	674	743	775	4.4
Revascularisation	40	47	79	79	94	94	92	101	105	99	111	126	7.6
Lumbar disc operation	67	77	88	79	76	77	74	72	68	62	64	60	– 2.1
Primary hip replacement	121	123	133	124	121	123	119	119	119	123	138	139	0.8
Primary knee replacement	90	94	106	105	117	126	126	130	137	143	167	185	6.0
Hysterectomy	629	617	609	580	617	670	689	673	640	599	605	555	– 0.4

**Figure 1:**
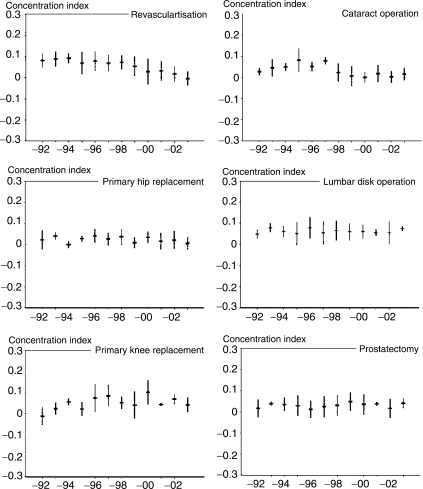
The development of socioeconomic differences in six elective operations between 1992 and 2003 among 25–84-year-old men; age-standardized concentration indices and their 95% confidence intervals.

Figure 1 presents CIs and their 95% confidence intervals for each procedure for each study year for men. In Figure 1, a concentration index with a negative value implies a distribution in which the worst-off income groups use relatively more services than the better-off groups, and a positive value a distribution in which the better-off use more services. For coronary revascularisations, relative income differences favoured the better-off groups in 1992, and the differences increased slightly in the beginning of the study period, but from the mid-1990s the differences decreased consistently. A linear trend of decreasing income differences was also found in regression analysis (*p* < 0.0001). For primary hip replacement operations, income group differences favouring the better-off were found for five study years. In the other years, the distribution of operations was income neutral, and no significant change in the differences was detected. For primary knee replacement operations, a pattern of relative income differences favouring the better-off was found throughout the 1990s. However, a trend of decreasing differences was detected in the early 2000s. Accordingly, no statistically significant linear trend was discernible. For cataract operations, a pattern favouring the better-off was found in the beginning of the study period, but the inequities declined after 1997, and no linear trend was detected. For lumbar disc operations, the CIs showed a steady pattern of differences favouring the better-off, with few changes in time. In only one of the study years were the results income neutral. For prostatectomy, few statistically significant differences were found in CIs.

**Figure 2:**
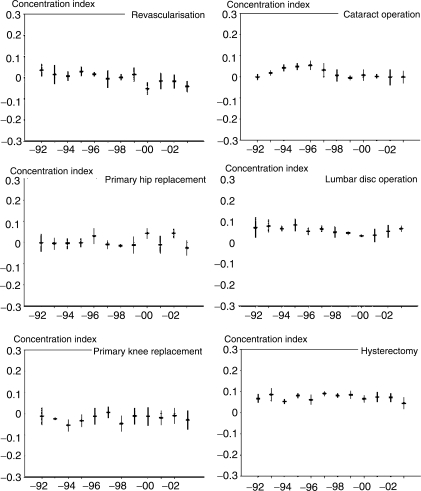
The development of socioeconomic differences in six elective operations between 1992 and 2003 among 25–84-year-old women; age-standardized concentration indices and their 95% confidence intervals.

Among women (Figure 2), the distribution of revascularisation operations favoured the better-off in the beginning of the study period, but the income group differences decreased steadily during the study period, resulting in a distribution favouring the worst-off income groups in the beginning of the 2000s. A linear trend was also found in the regression analysis (*p* = 0.0011). Primary hip replacement operations were relatively evenly distributed across income groups; in only two of the study years was a distribution favouring the better-off found. No significant linear trend was detected in the regression analysis. Primary knee replacements were relatively evenly distributed across income groups, and no linear trend could be detected in CIs through the study period. For cataract operations, a trend of increasing inequities favouring the better-off was found in the beginning of the study period. However, after 1996, a decreasing trend was detected, and by the end of the study period, the distribution of operations was income neutral. Accordingly, no significant trend could be fitted to the data. The distribution of lumbar disc operations favoured better-off income groups, but the trend varied in time: throughout 1990s, a trend of decreasing differences was found, and in the early 2000s, a trend of increasing differences. Hysterectomy showed a steady distribution of operations favouring the better-off, and the pattern was similar throughout the study period. No linear trend in differences was detected in the regression analysis.

## Discussion

We examined 12-year trends in socioeconomic inequalities in rates of elective surgery for coronary revascularisation, cataract extraction, hip and knee replacement, lumbar disc surgery, hysterectomy, and prostatectomy. Overall, income group differences were larger among men than among women; for some procedures, there was no clear relationship with income. Three clear patterns emerged, however: a decline in income-related inequality over time for coronary revascularisation; an increase at the start of the period, followed by a reduction in inequality, for cataract extraction and primary knee replacement among men; and positive relationship between income and treatment rates for lumbar disc surgery and hysterectomy.

Our results indicate that rates of elective surgery for several common conditions vary by income, even in a universal healthcare system. Our study was designed to compare the variation in access to care among procedures where inequalities appear to be most persistent, those where the patient makes a choice to present and the clinician has discretion over whether to treat. While similar inequalities in access to treatment have been identified in other countries [4–7], they seem to be more marked in Finland [1]. This reflects structural features of the healthcare system and factors influencing the behaviour of professional and patients that influence the socioeconomic patterning of treatment. The relative influence of these factors is likely to vary by procedure.

Our study employed individual register data on diagnosis and treatment, including referrals from the private sector and elective surgery undertaken privately. The Finnish Care Register has not been formally evaluated recently, but studies from the 1980s reported that about 95% of all discharges and 90–95% of surgical procedures were recorded in the register [13,14]. Several studies have assessed the validity of the Finnish Care Register diagnoses, and found that they compare satisfactorily with diagnoses made with standard criteria from the FINMONICA/FINAMI study in reporting hospital treatment for coronary heart disease [15,16].

In our study, the Finnish population aged 25–84 years formed the population at risk, because no individual-level data on need were available from the registers. Our estimates of relative differences in access to elective surgery, therefore, are likely to be conservative, since many of the conditions that we studied occur more frequently among lower socioeconomic groups [17]. During the study period, there was no evidence of any change in the distribution of need in relation to income.

Finnish data, however, do permit detailed analysis of inequalities in treatment rates, because individual data on socioeconomic status, including income, can be linked to health service use using unique personal identifiers. These data are reliable, and our previous studies have indicated that disposable income encapsulates the direct and indirect factors that affect access to planned care. These factors include the ability to pay for care, income as a proxy for established assets, and/or regular employment in a professional or managerial role [18].

We used CIs to compare the distribution of operations across income groups and to examine changes in the distribution over a 12-year period. This method enables all income groups to be included for examination of the gradient in inequality in access to care. It shows the direction and measure of (in)equity in distributions and allows modelling and quantitative measurement of distributional differences and changes over time. Similar methods have been used, e.g. to study differences in the distribution of health [12] and the use of outpatient services [19] between countries and between years in one country [20].

Several structural features of the health service and factors influencing the behaviour of professionals, managers and patients changed over the period that we studied. Together, these go some way to explaining our findings and those in other countries with similar experiences. At the beginning of the 12-year study period, the Finnish government decentralized health service funding and delegated decision-making to individual municipalities. The age and social structure of the municipalities, the income and infrastructure available to them to fund health services and the opportunities for collaboration with their neighbours varied across the country, perpetuating the risk of social and geographical inequalities in access to care [21].

The main tools available to the Ministry of Social Affairs and Health in Finland have been employed with varying levels of success to increase capacity and reduce inequalities in planned care. Benchmarking initiatives have been established, and clinicians have formalized working in clinical networks and developed integrated care pathways. These tools have been most effective where they have been supported by public and professional scrutiny from within and beyond Finland. Rates of coronary revascularisation, criteria for intervention and inequalities associated with previous treatment patterns, for example, have been subject to scrutiny across Europe and the OECD countries, highlighting the weight of evidence for the benefit of this approach.

The second pattern of treatment rates, an increase and then a decline in income-related inequities, was found particularly for cataract extraction, but also for primary knee replacement among men. This may also reflect national and international scrutiny of treatment practices [22] and advances in anaesthetic and surgical treatment enabling higher-risk patients to be treated. Australia, another country with a comprehensive health system, co-payments, and mixed public–private sector provision, found that, while older residents from less disadvantaged areas, and those who paid for private treatment, were more likely to undergo cataract extraction, there was no clear socioeconomic gradient, and by 2000–2001, the gap between major cities and remote and rural regions was narrowing [23]. While primary knee replacement among men followed a similar pattern in our study, in other countries where inequalities have been measured at the area level, the socioeconomic gap remains, e.g. in Canada [24] and England [5].

Potential explanations for the improvements in access to treatment in Finland identified above include behavioural changes at the individual and organisational levels that have facilitated more rapid expansion of services in recent years, and the development and implementation of widely agreed clinical guidelines, e.g. for hip and knee replacement [25].

The third pattern that we identified was the consistently pro-rich picture found on examination of the trends in lumbar disc operation and hysterectomy. Evidence-based guidelines on the use of these procedures reflect the requirement for caution, careful selection of patients to achieve optimum benefit, and alternatives to surgical intervention [26,27]. In both cases, these have spread into routine practice more extensively in Finland than in other OECD countries, perhaps because of easier access to private specialists favouring surgery.

Although the proportion of hospital inpatient treatment in the private sector has been relatively low in Finland, private ambulatory care and assessments affect access to some elective surgical procedures. Referrals from the private sector are also an important source of differential access. According to the Finnish Care Register in 2003, 80% of patients undergoing cataract extraction were referred by a private practitioner. For hysterectomy, the proportion was around 50%, and for primary hip and knee operations, approximately one-third. In contrast, for coronary revascularisation, one of the interventions for which evidence of inequalities was reducing, the proportion of private sector care was about 7%.

The existence of a parallel system in which additional payments provide a choice of provider increases income differences in access to assessment and treatment. In Denmark, it also increased the share of health service expenditure among more affluent groups [28]. A similar scheme to enable employers to purchase additional elective care for their employees was ruled out in the Netherlands because of its potential to increase health inequalities [29].

## Conclusions

This study identified varied patterns but persistent socioeconomic differences in access to elective surgery for seven common, chronic problems. While there were some positive findings that could be attributed to changes in clinical and organisational practice, particularly those associated with expansion of eligibility and provision, several structural features of the Finnish healthcare system have an impact on maintaining inequity in access to these procedures and help to explain the greater socioeconomic differences found in Finland as compared to other Nordic countries.
